# Estimating utility value for female genital mutilation

**DOI:** 10.1186/s12889-020-08947-4

**Published:** 2020-05-29

**Authors:** Cyrus Alinia, Bakhtiar Piroozi, Fariba Jahanbin, Hossein Safari, Amjad Mohamadi-Bolbanabad, Ali Kazemi-Karyani, Ghobad Moradi, Fariba Farhadifar, Mohammad Ebrahimi

**Affiliations:** 1 Department of Health Management and Economics, School of Public Health, Urmia University of Medical Sciences, Urmia, Iran; 2 Reproductive Health Research Center, Urmia University of Medical Sciences, Urmia, Iran; 3 Social Determinants of Health Research Center, Research Institute for Health Development, Kurdistan University of Medical Sciences, Sanandaj, Iran; 4 Health Promotion Research Center, Iran University of Medical Sciences, Tehran, Iran; 5 Research Center for Environmental Determinants of Health, Health Institute, Kermanshah University of Medical Sciences, Kermanshah, Iran

**Keywords:** Female genital mutilation, Circumcision, Disease utility, DALY, Women’s health

## Abstract

**Background:**

Female genital mutilation/cutting (FGM/C) is a clear violation of women’s rights and can have adverse and irreversible health effects as well. Worldwide, more than 200 million women and girls have undergone FGM/C. Utility value of FGM/C has not been estimated yet, so we designed this study to extract the health utility value of FGM/C for the first time in the world.

**Methods:**

In a cross-sectional study in Iran, 125 girls and women who underwent FGM/C procedure were examined by the trained midwives in order to determine its type. In addition, a questionnaire was completed for identifying the socio-demographic factors and extracting the health utility of these individuals. Health utility was measured using Time Trade-off method and also to determine the effects of the socio-demographic factors on the health utility a two-limit censored regression model was applied.

**Results:**

The mean and median of the health utility of women with FGM/C were 0.971 (SE: 0.003) and 0.968 (IQR: 1–0.95), respectively. Number of non-traders was 58 (46.4%) who reported perfect health utility. However, the mean of health utility among traders was 0.946 (SE: 0.002). Only type 1 (Clitoridectomy) and type 2 (Excision) FGM/C were seen in this study. Women with Type 1 FGM/C had significantly lower health utility value (Mean: 0.968, Median: 0.957) than their type 2 counterparts (Mean: 0.987, Median: 1.00). Moreover, women in the age group of 31–45 years (Mean: 0.962, Median: 0.956), single (Mean: 0.950, Median: 0.954), divorced (Mean: 0.951, Median: 0.950), employed (Mean: 0.959, Median: 0.956), and with supplementary insurance (Mean: 0.962, Median: 0.950) had significantly lower health utility than their counterparts.

**Conclusion:**

FGM/C affects physical and psychological well-being of these individuals, resulting in a lack of personal and marital satisfaction, which ultimately leads to a 3% reduction in their health related quality of life. Therefore, preventing from this practice is very important and should be considered by health system policy makers more than before.

## Background

Female Genital Mutilation/Cutting (FGM/C), which is considered as a global concern, is defined by World Health Organization (WHO) as “all procedures that involve the partial or total removal of external genitalia or another injury to the female genital organs for non-medical reasons” [1]. The practice, which is also considered as violence against women, is prevalent in 30 African and some Middle Eastern and Asian countries and threatens the life of about 3.6 million girls and women annually [2]. Estimates have shown that more than 200 million girls and women have experienced this counter-human rights procedure [3].

The highest prevalence of FGM/C is seen in African countries such as Somalia (98%), Guinea (96%), Djibouti (93%), and Egypt (91%) [4]. It may also be seen among some ethnic groups and immigrants living in developed European and North American countries and Australia [5]. Although there are no official and comprehensive statistics on the prevalence of FGM/C in Iran, the results of few studies have shown that the prevalence of the practice varies by region and is mainly concentrated in the western and southwestern provinces (Hormozgan, Khuzestan, Bushehr, Kurdistan, and Kermanshah [6–8] which are mostly inhabited by Sunni Muslims. The prevalence of the FGM/C is reported to be between 55.5 and 70% primarily in rural of these areas [9, 10].

Researchers have divided the causes behind practicing FGM/C into five groups: psychosexual, sociological and cultural, hygienic and aesthetic, religious, and socio-economic [11]. Some studies have shown that the main reasons for performing this practice in Iran are the preservation of traditions, cleanliness, religious recommendations, and control of sexual desire, respectively [8, 9]. These factors have turned FGM/C, as an element for inequality and violence against women, into a social norm or conviction [12]. However, so far, not only no hygienic or unsanitary benefits have been reported for this procedure, but also numerous studies have shown its increasing and long-term adverse effects. Its immediate effects include shock, severe pain, extensive bleeding, swelling, damage or infection of the genital area, fever, and, in some cases, even death. Long-term effects of FGM/C include urinary problems, vaginal problems (such as discharge, infection and itching), menstrual problems, scar tissue and keloid, sexual dissatisfaction, high-risk pregnancy, psychological issues (depression, anxiety, post-traumatic stress disorder, low self-esteem and self-confidence), social isolation, and sanitation problems [2, 13–16]. However, the health-related quality of life of women living with FGM/C has not been quantitatively measured. Therefore, in this study, we aimed to measure its utility value among different socio-demographic groups in order to objectively determine the total effects of FGM/C on health-related quality of life of women and girls.

## Methods

### Study population

This cross-sectional and multicenter study was conducted in 2018 in Kermanshah province, west of Iran. One hundred twenty-five females aged over 16 who had experienced the FGM/C participated in the study after giving their written consent. The samples were selected by non-random consecutive sampling method from the girls or women referred to the midwifery offices for examination. Next, they were examined by trained midwives, and a questionnaire was completed for them. An obstetrician examined these women for having FGM/C and identified its type.

Type 1 FGM/C (Clitoridectomy) means removing part or all of the clitoris; type 2 (Excision) is defined as removing part or all of the clitoris and the labia minora, with or without removal of the labia majora; type 3 (infibulation) is narrowing of the vaginal opening by creating a seal, formed by cutting and repositioning the labia; and type 4 (other harmful procedures) means any injury to the female genitals, including pricking, piercing, cutting, scraping or burning the area. The females who did not have any illness, disorder, and psychological disability, except FGM/C entered the study.

### Measurement of utility value

Time trade-off (TTO) approach has been used to measure the health-related utility of FGM/C. In this approach, the researcher asked the participants: how many years in the current health state (X) you would be willing to lose in order to regain full health. The number of these years changes to the degree that the individual has to subjectively stay indifferent between their health state in remaining future years (Y) and perfect health state in a shorter period of time and sees their value both the same. The amount (x/y) is presented as disutility and the result of formula 1- (x/y) indicates the health utility of each individual. In all cases, in the first question, the value of x was considered four years. If the interviewees agree/disagree, this amount could be increased/decreased, and it continued until reaching the indifference point. Life expectancy at birth for Iranian women was considered 79 years.

The best and the worst health state of the individual measured by TTO method equals zero and one, respectively. It gets zero if the individual is not willing to lose any years of their life, and it gets one if the individual is willing to lose all their remaining years of life to fix the FGM/C problem.

### Measurement of socio-demographic characteristics

After determining the type of FGM/C, questions about socio-demographic factors were asked from the participants by the midwife. These questions included determining the age (grouped in three classes: 19–30, 31–45, and over 46), marital status (single, married and widowed/divorced), education (illiterate, high school graduate, and university graduate), employment status (unemployed, employed, and housewife), standardized monthly household expenses (monthly household expenditure is divided by its household dimension), and having basic and supplementary health insurance.

The two-limit Tobit linear regression model was used in order to determine the effects of socio-demographic factors on the level of health utility among women with FG/CM. In this model, given that the dependent variable is assumed to have a normal distribution; therefore, we considered the logarithmic value of health utility as a dependent variable and factors of age (years of life); type of FGM/C (1: type 2 and 0: type 1); marital status (1: married and 0: non-married); education (number of years of education); occupation (1: employed and 0: non-employed); and standardized monthly household expenditure (average monthly spending per person) as independent variables. To determine the predictive variables in this model, some univariate logistic regressions were performed for all socio-economics factors. Only parameters were selected that had a statistically significant association with the dependent variable at 0.05 error level. Pearson test for scale variables and Spearman test for categorical variables were used to identify the autocorrelation problem.

### Statistical analysis

The distribution of studied women among socio-demographic groups is represented by number and percentage. For each of these socio-demographic sub-groups, the amount of measured utility has been reported with mean, standard error, median, and interquartile range.

Due to the non-normal distribution of the utility values, Kruskal Wallis (for groups with more than two subgroups) and Mann-Whitney (for groups with two subgroups) were used to measure the significant difference between the mean values for subgroups of each socio-demographic group. The exchange rate used for conversion is 1 USD = 120,000 Iranian Rials. The significance level was set at a *p*-value < 0.05. All statistical analyzes were performed using STATA version 13 (Stata Crop LP, College Station, TX, USA).

### Ethical considerations

The Research Deputy of Kurdistan University of Medical Sciences approved this study. Before the study, all participants gave written informed consent to participate in the study. The researchers adhered to the tenets of the Declaration of Helsinki in the implementation of all stages of the study.

## Results

A total of 125 women and girls who had a history of FGM/C participated in this study. The mean age of these participants was 35.74 years (ranged 19–55). The participants included 10 (8%) single women, 17 (13.6%) divorced / widowed, and the rest were married. The distribution of first and second type of FGM/C among the participants was 104 (83.2%) and 21 (16.8%), respectively (Table 1).

**Table 1:** Utility values of Female Genital Mutilation among different socio-economic groups

Socio-Demographic Factors & FGM/C types	Number (%)	Utility value		*P*-value
		Mean (SE)	Median (IQR)	
Total	125 (100)	0.971 (0.003)	0.968 (1–0.950)	
Age				0.001
Young (19–30 years)	42 (16.0)	0.978 (0.004)	1.000 (1–0.968)	
Not Young (31–45 years)	63 (50.4)	0.962 (0.003)	0.956 (1–0.936)	
Middle age (46–55 years)	20 (33.6)	0.985 (0.005)	1.000 (1–0.950)	
Marital Status				0.003
Single	10 (8,0)	0.950 (0.006)	0.954 (0.957–0.936)	
Married	98 (78,4)	0.977 (0.003)	1.000 (1–0.956)	
Divorced	17 (13.6)	0.951 (0.001)	0.950 (0.950–0.950)	
FGM/C types				0.051
Clitoridectomy	104 (83.2)	0.968 (0.013)	0.957 (1–0.943)	
Excision	21 (16.8)	0.987 (0.014)	1.000 (1–0.968)	
Education				0.009
Illiterate	39 (31.2)	0.981 (0.005)	1.000 (1–0.956)	
High school	32 (25.6)	0.967 (0.004)	0.950 (1–0.950)	
University	54 (43.2)	0.967 (0.004)	1.000 (1–0.956)	
Job				0.023
Employed	16 (12.8)	0.959 (0.027)	0.956 (0.979–0.941)	
Unemployed	33 (26.4)	0.963 (0.004)	0.950 (1–0.950)	
Housewife	76 (60.8)	0.978 (0.030)	1.000 (1–0.956)	
Having basic insurance				0.203
Yes	105 (84,0)	0.970 (0.029)	0.958 (1–0.950)	
No	20 (16,0)	0.979 (0.06)	0.992 (1–0.968)	
Having Supplementary insurance				0.024
Yes	34 (27.2)	0.962 (0.028)	0.950 (1–0.950)	
No	91 (72.8)	0.975 90.028)	1.000 (1–0.952)	
Standardized household’s monthly cost				0.077
Lowest (< 50 USD)	111 (88.8)	0.970 (0.029)	0.968 (1–0.950)	
Highest (> 50 USD)	14 (11.2)	0.984 (0.026)	1.000 (1–0.956)	

*SE* Standard error, *IQR* Interquartile range

The results of the Tobit regression analysis are presented in Table 2. As shown here, FGM/C disutility had a significant positive relationship with the factors of having a job, age, income and being single.

**Table 2:** Estimation results of the two-limit Tobit model of utility value for Female Genital Mutilation

Variables	Coefficient	t statistics	***P***-value
Age	0.008	2.45	0.021
FGM/C types	0.037	2.79	0.096
Marital status	0.005	2.61	0.014
Education	−0.003	−0.60	0.553
Job	0.007	2.20	0.030
Standardized monthly expenditure	0.035	2.14	0.034

## Discussion

The FGM/C procedure, which acts as a violation of women’s rights and is considered as violence against women, imposes much physical, psychosocial and social harm on women and girls, and reduces their health-related quality of life. For the first time, the current study measured the utility value of FGM/C for different socio-demographic groups. The results indicated that women/girls living with FGM/C had an average health utility of 0.971 (SE: 0.003) with a median of 0.968 (IQR: 1–0.95). In other words, they lost about 3% of their Health-Related Quality of Life (HRQoL) due to FGM/C, on average.

Generally, the results of the regression model and univariate analysis were highly consistent, except for the education level. These findings confirm that the value of FGM/C disutility is positively and significantly associated with age, income level, having a job, and not having a husband. Univariate analysis indicates a statistically significant association between FGM/C disutility and education years, but this relationship is not confirmed by multivariate analysis. Of course, these results need to be interpreted with caution, which are discussed below.

The utility value, which subjectively represents the sum of the effects of FGM/C on women’s health, was statistically significantly lower for single (Mean: 0.950, Median:0.954) and divorced women (Mean: 0.951, Median: 0.950) compared to married women (Mean: 0.977, Median:1.00). In other words, single and divorced women experienced more reduction in their HRQoL than married women. This finding is confirmed in both univariate and multivariate analysis models. One possible explanation for this finding is that FGM/C practice delayed marriage for the circumcised single women, as the mean age of them was significantly higher than their married counterparts (38.2 vs. 33.8 years). Besides, it could conceivably be hypothesized that FGM/C, due to its psychosexual problems [11], is a crucial factor in the decision to divorce and thereby increasing the disutility of the practice, which is an important issue for future research.

This negative effect may vary in different countries and cultures, as in countries such as Nigeria, Somalia, and Sudan, girls who have performed FGM/C have a higher chance of finding a partner and having a timely marriage [17, 18]. While in Guinea, having or not having FGM/C, did not have any effect on the chance of getting married [19]. While Iran has a much more open society and prevalent interracial marriage than African and other Middle East countries, unlike these countries, performing the FGM/C does not necessarily increase the chance of marriage. FGM/C practice, which is performed only among a part of Sunni minority living in the western border areas of Iran, is generally considered as an abominable and unacceptable practice from society’s point of view. So, performing this practice even may decrease the marriageability. Nonetheless, a definitive answer to this requires more in-depth studies [20]. Another reason can be related to the perception of single women of marital restrictions of FGM/C, such as intercourse pain, less sexual pleasure and related bleeding. Since women from less developed societies may not experience these restrictions before marriage, they may over-estimate them [17]. The divorced women may also attribute their separation and failure in life to FGM/C.

Another important result of this study was that women in the 31–45 age group statistically significantly reported a lower HRQoL (Mean: 0.962, Median: 0.956) than others. The reason could explain this finding is that middle-aged women are more likely to have experienced the adverse effects of the FGM/C than their younger counterparts, and they also have higher expectations of the quality of their sexual relations than their adult peers. However, further analysis of the data showed that the middle-aged group had the highest divorce rate than others, accounting for 69% of all divorces. This result could support the theory, as mentioned earlier, that FGM/C could be a risk factor for divorce. This finding is in line with the results of other studies [21–24].

Unexpectedly findings showed that employed subjects reported less health utility than unemployed women and housewives (Mean: 0.959, Median: 0.956). It is difficult to explain this result, but with more analysis, we find that the average years of education in this group were significantly higher than in other groups so that all 16 members of this group had a university degree. Therefore, the difference in their level of disutility may be more due to their level of education, not their employment status. To study the association between employment factor and HRQoL of women with FGM, a larger sample size that enables us to control factors such as age, marital status, and degree of education are required, and this study is not able to give an accurate answer in this regard.

In this study, only FGM/C types 1 and 2 have been seen, and due to the very low prevalence of other more severe types of the practice, we have not found them [8]. Although we know that FGM/C type 2 is much more invasive, painful, and has more side effects than type 1, surprisingly, our findings showed that FGM/C type 1 had higher disutility value than another. Of course, it should be noted that this difference was not statistically significant in any of the analytical models. Since the group with FGM/C type 2 had higher age means than type 1 (37.47 vs. 27.14) and were completely different in terms of marital status (all participants who have never been married or divorced were in type 1 group), we are not allowed to compare their health utility values. Therefore, to more accurately compare the effects of type of FGM/C on HRQoL, a larger sample size with the same age strata is required for all types of FGM/C.

Fifty-eight participants of the study (46.4%) were not willing to lose any time to regain their usual genital condition, and they reported a full health utility. These are referred to as non-traders. All of them were married, and more than 81% of them were housewives. These people, on average, had a larger household size with a monthly household expenditure below the average. Therefore, it can be concluded that non-traders were less influenced by the effects of FGM/C, and were able to live their lives following the community conditions and their expectations, so they did not complain about it. The mean health utility for traders was 0.946 (95% CI: 0.943–0.950). In other words, women, who have been hurt in their personal and family life due to FGM/C, lose 5.4% of their HRQoL.

We know that the effects of FGM/C, which violates women’s rights, are not limited to their health and HRQoL, but their individual and social lives. According to the findings, if we consider only the HRQoL effects, FGM/C can be compared with diseases such as non-severe hypoglycemic, mild primary dysmenorrhea, skin neoplasm, myopia, and otitis media associated with pain. On average, these all result in a 3% loss of HRQoL in individuals. Subgroups such as single or middle-aged women with FGM/C, who are affected by this procedure more than others, have disutility almost equal to mild gastroenteritis. Of course, since the FGM/C procedure is mainly performed during the childbirth or childhood, it has life-time effects and imposes more disease burden than the diseases as mentioned earlier (Table 3). If we assume that a girl performs FGM/C in her first year of life, with a discount rate of 3% and a life expectancy of 75 years, its burden of disease is equal to 0.87 years.

**Table 3:** Comparison of FGM’s utility value with other diseases

Diseases	Mean of utility value	Reference
Hypoglycemic- Non-severe daytime event	0.972	[25]
Hypoglycemic- Non-severe nocturnal event	0.977	[25]
Stable schizophrenia	0.919	[26]
Mild Primary Dysmenorrhea	0.970	[27]
Skin neoplasm of uncertain behavior	0.970	[28]
Epilepsy	0.920	[29]
Myopia	0.970	[30]
Otitis media with pain	0.970	[31]
Moderate gastroenteritis	0.940	[31]

The burden of FGM/C is unnecessary and avoidable, provided that governments take indigenous and effective initiatives and measures to prevent the FGM/C procedure. To develop domestic protocols, the main reasons for performing FGM/C should be extracted in each community and should be considered as a basis for identifying prevention strategies.

Numerous studies have shown that the social factors that determine the performance of FGM/C vary from country to another, which requires different policies to control this practice. Snow et al. introduced ethnicity, age, religion, and education as the most critical social predictors of FGM/C in Nigeria [32], which had a high consistent with Ofori-Fosu’s findings in Ghana [33]. Bogale et al. concluded that the main reasons for the perpetuation of this practice in Ethiopia were religion, safeguarding virginity, tradition, and social values, respectively [34]. Ouedraogo, meanwhile, sees social pressure as the main reason for the decision to practice excision in Burkina Faso households [35]. According to Afifi’s findings in Egypt and Satti et al. In Sudan, low levels of education were the main culprits for FGM, which with the increase in the level of education, women’s desire to continue this practice has significantly decreased for their daughters [36, 37].

Of course, all prevention programs require intersectoral collaboration and should include activities such as health education to parents, passing national laws, standing against some meaningless social norms and traditional beliefs, encouragement of mothers and girls to educate, and getting help from religious missionaries [38, 39]. Nowadays, one of the new barriers to eliminate FGM/C in some countries, especially African countries, is medicalization. Doctors, nurses, and some other health workers claim that they can perform this procedure on sanitary conditions, so they give FGM/C legitimacy. It not only does not help the prevention of FGM/C, but it also encourages people to do it. Therefore, announcing that performing FGM/C is against the law can also be a useful step to reduce the incidence of this procedure [40, 41].

The strength of the current study was to examine FGM/C victims by trained midwives to determine the degree of FGM/C. However, the results of the present study should be interpreted in light of its limitations. First, we did not include types 3 and 4 of FGM/C; therefore, our results can only be generalized to women with types 1 and 2. Given the fact that the more severe types of this disorder impose more long-term adverse effects on the individuals and create more family and personal problems for them, it may also generate greater health disutility. Therefore, it is highly recommended that the disutility of FGM/C types 3 and 4 should be extracted in other studies. Second, the value of health disutility of FGM/C seems to be very much influenced by the culture of the community, so if the restrictions imposed by FGM/C prevent women from meeting their personal and social expectations, it can raise women’s dissatisfaction and create more disutility. Therefore, the results of this study are not necessarily the same in different societies and communities.

## Conclusion

Iranian women undergoing FGM/C types 1 and 2 lose an average of 3% of their HRQoL due to their health problems. Of course, this figure for non-traders and traders is estimated as zero and 5.5%, respectively. Therefore, the epidemiological burden of FGM/C is very considerable, and it is recommended that the countries involved in this social and health challenge develop prevention protocols for FGM/C and stick at them seriously.

## Data Availability

The datasets generated and/or analyzed during the current study are not publicly available for confidentiality reasons since individual privacy could be compromised but are available from the corresponding author on reasonable request.

## Abbreviations

FGM/C: Female Genital Mutilation/ Cutting
WHO: World Health Organization
TTO: Time trade-off
HRQoL: Health-Related Quality of Life
